# Genetic Reassortment in a Child Coinfected with Two Influenza B Viruses, B/Yamagata Lineage and B/Victoria-Lineage Strains

**DOI:** 10.3390/v16060983

**Published:** 2024-06-19

**Authors:** Yoko Matsuzaki, Kanetsu Sugawara, Yuko Kidoguchi, Yoko Kadowaki, Yoshitaka Shimotai, Yuriko Katsushima, Fumio Katsushima, Shizuka Tanaka, Yohei Matoba, Kenichi Komabayashi, Yoko Aoki, Katsumi Mizuta

**Affiliations:** 1 Department of Infectious Diseases, Yamagata University Faculty of Medicine, Yamagata 990-9585, Japan; ksugawar@med.id.yamagata-u.ac.jp (K.S.); keikoyuko@med.id.yamagata-u.ac.jp (Y.K.); yokocadow@med.id.yamagata-u.ac.jp (Y.K.); yoshimo@med.id.yamagata-u.ac.jp (Y.S.); 2 Katsushima Pediatric Clinic, Yamagata 990-2461, Japan; naohiro-yurikatsu@s3.dion.ne.jp (Y.K.); fkatsu@yf6.so-net.ne.jp (F.K.); 3 Department of Microbiology, Yamagata Prefectural Institute of Public Health, Yamagata 990-0031, Japan; tanakashiz@ypch.gr.jp (S.T.); matobay@pref.yamagata.jp (Y.M.); komabayashike@pref.yamagata.jp (K.K.); aokiyok@pref.yamagata.jp (Y.A.); mizutak@pref.yamagata.jp (K.M.)

**Keywords:** influenza B virus, reassortment, reassortant, coinfection

## Abstract

We identified a child coinfected with influenza B viruses of B/Yamagata and B/Victoria lineages, in whom we analyzed the occurrence of genetic reassortment. Plaque purification was performed using a throat swab specimen from a 9-year-old child, resulting in 34 well-isolated plaques. The genomic composition of eight gene segments (HA, NA, PB1, PB2, PA, NP, M, and NS genes) for each plaque was determined at the lineage level. Of the 34 plaques, 21 (61.8%) had B/Phuket/3073/2013 (B/Yamagata)-like sequences in all gene segments, while the other 13 (38.2%) were reassortants with B/Texas/02/2013 (B/Victoria)-like sequences in 1–5 of the 8 segments. The PB1 segment had the most B/Victoria lineage genes (23.5%; 8 of 34 plaques), while PB2 and PA had the least (2.9%; 1 of 34 plaques). Reassortants with B/Victoria lineage genes in 2–5 segments showed the same level of growth as viruses with B/Yamagata lineage genes in all segments. However, reassortants with B/Victoria lineage genes only in the NA, PB1, NP, or NS segments exhibited reduced or undetectable growth. We demonstrated that various gene reassortments occurred in a child. These results suggest that simultaneous outbreaks of two influenza B virus lineages increase genetic diversity and could promote the emergence of new epidemic strains.

## 1. Introduction

Seasonal epidemics of influenza B virus occur in humans, similar to those of influenza A virus. Influenza B virus is genetically and antigenically divided into two lineages, B/Victoria and B/Yamagata, which are named after the reference strains B/Victoria/2/87 and B/Yamagata/16/88, respectively [1]. Two influenza B lineages co-circulate among humans globally, along with influenza A subtypes A/H1pdm09 and A/H3. This has necessitated the development of quadrivalent influenza vaccines to protect against both influenza B lineages [2]. If outbreaks of different lineages occur at the same time, individuals could be simultaneously infected with two influenza B lineages.

Influenza B virus is a member of the Orthomyxoviridae family, along with influenza A, C, and D viruses. It contains a single-stranded, negative sense segmented genome. This genome consists of eight RNA segments encoding two surface glycoproteins [hemagglutinin (HA) and neuraminidase (NA)], three polymerase proteins (PB2, PB1, and PA), a nucleoprotein (NP), a matrix (M) protein, and a nonstructural (NS) protein. Upon coinfection of the same cell by two viruses, the segmented genomes undergo reassortment, characterized by segment exchange between the viruses. Studies have revealed the importance of such reassortment in the evolution of influenza viruses [3,4,5]. It has also been shown that several historical pandemics were caused by intersubtypic reassortments between avian, swine, and/or human influenza A viruses [6,7]. Unlike influenza A virus, influenza B virus predominantly circulates only in humans and does not consist of different subtypes, meaning that no potentially pandemic-causing intersubtypic reassortment called antigenic shift can occur.

Phylogenetic analyses of influenza B viruses have indicated that, since 2000, extensive reassortment events have occurred both between and within the B/Victoria and B/Yamagata lineages [3,8,9,10,11,12,13,14,15]. However, to the best of our knowledge, no reports have been published on humans coinfected with two distinct influenza B viruses between which genetic reassortment occurred. Nonetheless, in one study using a ferret model of human influenza, it was shown that viral interference between influenza B virus lineages occurred at challenge intervals of 3 days, while coinfections were found to be rare [16]. In a part of Japan, where two influenza B lineages co-circulated, we encountered a child coinfected with these two lineages in 2016. Here, we demonstrate that genetic reassortment occurred between them within this child.

## 2. Materials and Methods

### 2.1. Virus Isolation

As part of the National Epidemiological Surveillance of Infectious Diseases, Japan, patients with acute respiratory illnesses who visited two pediatric outpatient clinics in Yamagata Prefecture underwent the collection of respiratory specimens. Informed consent was obtained from each patient or patient’s guardian(s). The specimens were transferred to the Yamagata Prefectural Institute of Public Health, where they underwent viral isolation as a part of routine epidemic surveillance. Isolation was carried out via the microplate method as previously described [17]; all influenza virus strains were isolated using Madin–Darby canine kidney (MDCK) cells. The hemagglutination inhibition (HI) test using antisera against vaccine strains with 1.0% guinea pig erythrocytes was used to identify types A/H3, A/H1pdm09, B/Yamagata, and B/Victoria. HI titer was expressed as the reciprocal of the highest antibody dilution that completely inhibited hemagglutination.

### 2.2. Plaque Purification

Plaque purification was performed using MDCK cells. A specimen was centrifuged at 1000 rpm for 15 min, after which 100 μL of the obtained supernatant was inoculated into each well of a six-well plate containing monolayers of MDCK cells. After incubation at 37 °C for 60 min, the cells were overlaid with Eagle’s Minimum Essential Medium (MEM) containing 0.8% agar and 2 μg/mL TPCK-trypsin and incubated at 37 °C. Three days after infection, a second overlay medium containing 1% neutral red was added, followed by incubation for a further 24 h. After picking up well-isolated plaques, each was suspended in 300 μL of MEM. A total of 100 μL of this was used for sequencing, while the rest was used for further inoculation of MDCK cells. Viruses grown in MDCK cells were then used for a growth experiment, sequencing, and phylogenetic analysis.

### 2.3. Nucleotide Sequencing and Phylogenetic Analysis

Viral RNA was extracted from 100 μL of viral fluid using the QIAamp Viral RNA Mini kit (Qiagen, Hilden, Germany). The complete coding region of eight gene segments (HA, NA, PB1, PB2, PA, NP, M, and NS genes) was amplified by reverse-transcription PCR and sequenced using a BigDye Terminator v3.1 cycle sequencing kit (Life Technologies, Carlsbad, CA, USA) and an ABI Prism 3130 sequencer (Applied Biosystems, Foster City, CA, USA). The maximum-likelihood method, based on a general time reversibility model with 1000 bootstrap replicates, was used to construct phylogenetic trees using MEGA6 software [18]. The GenBank accession numbers for nucleotide sequences determined in this study are LC811801-LC811896, while those of the other sequences used for phylogenetic analysis are listed in Supplementary Table S1.

### 2.4. Viral Growth Kinetics

MDCK cells were infected with the indicated viruses at a multiplicity of infection of 0.001. Sixty minutes later, they were washed and incubated in the presence of 2 μg/mL TPCK-trypsin at 37 °C. The culture medium supernatants were harvested at 18, 24, 48, 72, and 96 h after infection. Viral titers were determined by plaque assays with MDCK cells. Briefly, serially 10-fold-diluted supernatants were inoculated onto MDCK cell monolayers cultured in a 12-well plate. After incubation at 37 °C for 60 min, cells were overlaid with MEM containing 1% Avicel and 2 μg/mL TPCK-trypsin and incubated at 37 °C. Three days after infection, the cells were fixed and incubated with an antiNP monoclonal antibody as the primary antibody and goat antimouse IgG conjugated with horseradish peroxidase as a secondary antibody. Subsequently, the visualization and counting of plaques were performed using TrueBlue (SeraCare, Milford, MA, USA). Experiments were performed in triplicate, with the results expressed as mean ± standard deviation.

## 3. Results

### 3.1. Antigenicity of Influenza B Viruses Isolated in Yamagata, Japan, in 2016

**Figure 1:**
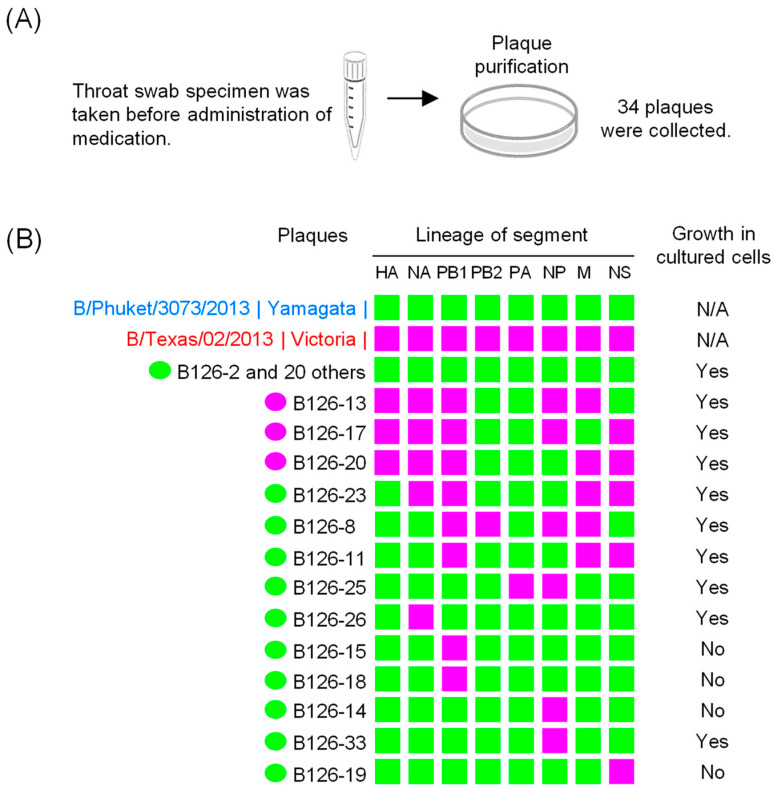
Summary of a specimen (B126) collected from a patient coinfected with two influenza B virus lineages. Plaque purification (**A**). Genomic composition of the 34 plaques at the lineage level (**B**). The color of each lineage matches that of the reference strains, B/Phuket/3073/2013 (all segments are B/Yamagata) and B/Texas/02/2013 (all segments are B/Victoria). N/A: not applicable.

A total of 82 influenza B viruses were isolated in Yamagata, Japan, between January and August 2016. Upon analyzing the antigenicity using antisera against the 2015–2016 vaccine strains, it was found that 62 strains belonged to the B/Yamagata lineage (B/Phuket/3073/2013-like), while 20 belonged to the B/Victoria lineage (B/Texas/02/2013-like). Only one strain, B/Yamagata/126/2016 (B126) virus, was found to have antigenicity reflecting both lineages, as shown in Table 1. This B126 virus was counted as belonging to the B/Yamagata lineage. It was isolated using MDCK cells from a throat swab taken from a 9-year-old boy with no underlying medical conditions. He visited a clinic on March 2, 2016, and a throat swab specimen was taken before administration of medication (Figure 1A).

### 3.2. Plaque Purification

To confirm that this patient had been coinfected with the two viruses, we performed plaque purification using the original swab specimen of the B126 virus collected on March 2. We collected 34 well-isolated plaques and performed direct sequencing to determine the lineage of each segment. Among the 34 plaques, 21 (61.8%) had B/Phuket/3073/2013-like sequences (B/Yamagata) in all segments (Figure 1B), while the other 13 (38.2%) were reassortants with B/Texas/02/2013-like sequences (B/Victoria) in 1–5 of the 8 segments. Only 1 of 34 plaques (2.9%) had a PB2 or PA segment with B/Victoria lineage sequences (Table 2). The PB1 segment had the highest number of B/Victoria lineage sequences; that is, 8 (23.5%) out of 34 plaques had PB1 segments with such sequences.

### 3.3. Phylogenetic Analysis

We created a phylogenetic tree and performed subsequent growth experiments using viruses grown by the propagation of 34 plaques in MDCK cells. No growth in MDCK cells was observed for two plaques with B/Victoria lineage sequences only in the PB1 segment, one plaque with B/Victoria lineage sequences only in the NP segment, and one plaque with B/Victoria lineage sequences only in the NS segment (Figure 1B).

**Figure 2:**
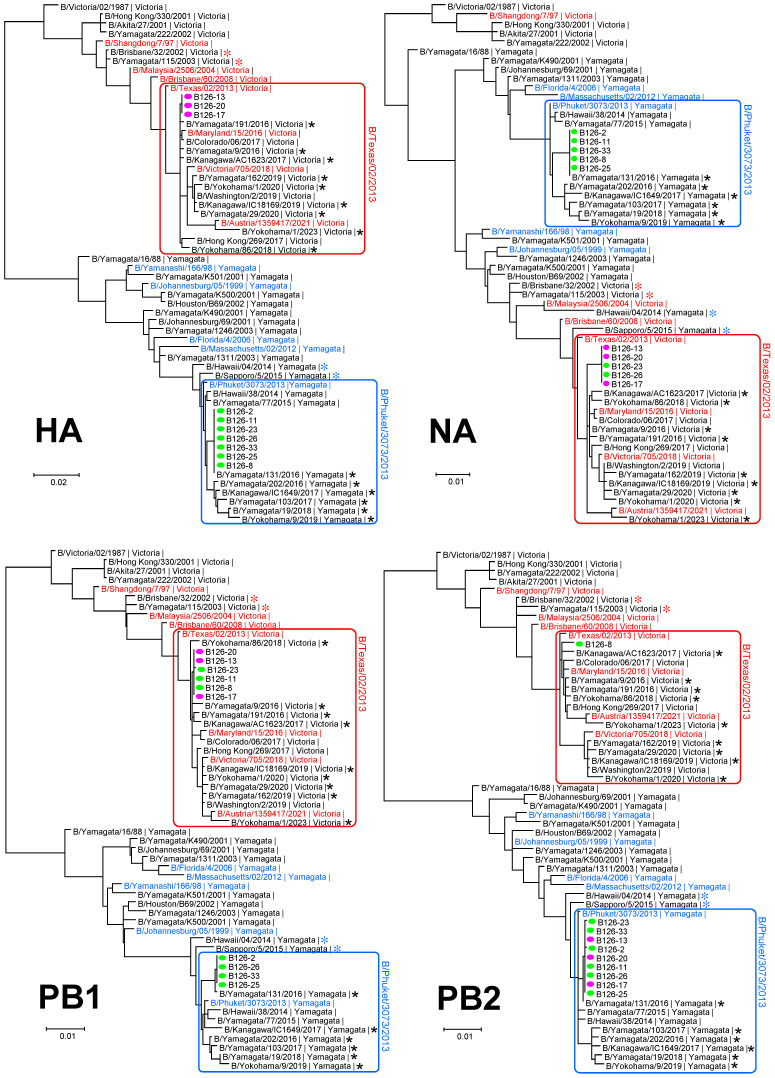

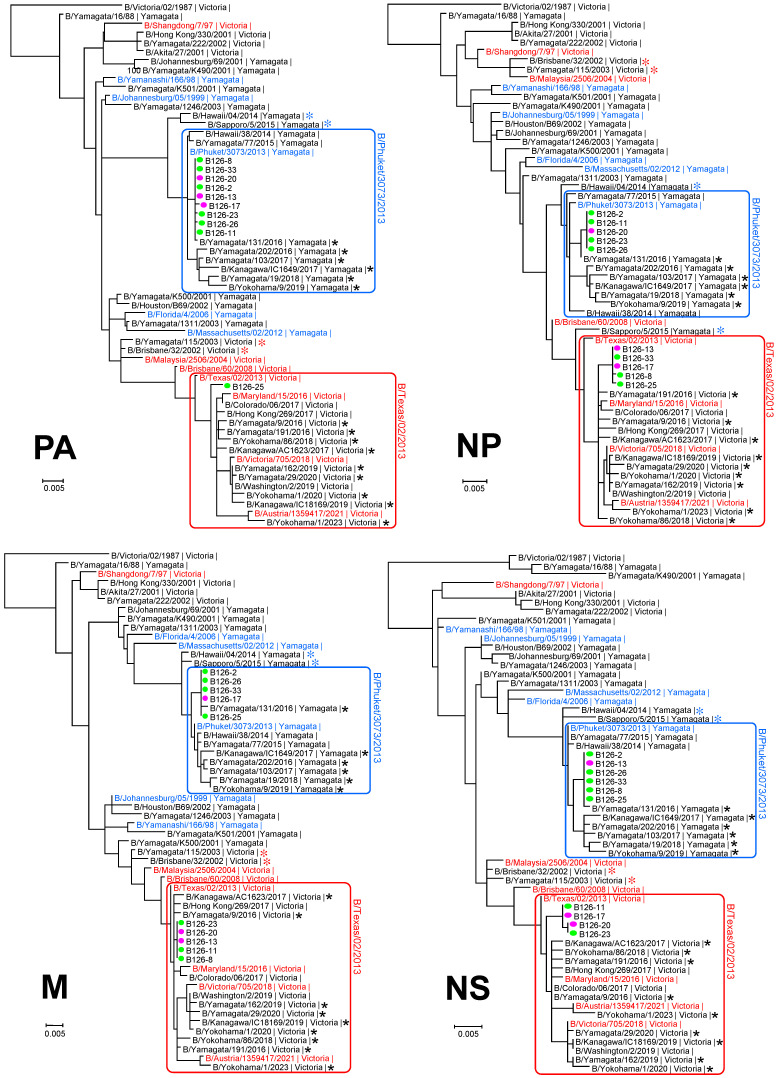
Phylogenetic analysis of the eight segments of reassortants derived from a B126 specimen collected from a patient coinfected with two influenza B virus lineages. The HA lineages of B126 viruses are indicated by magenta ovals for B/Victoria and green ovals for B/Yamagata. The successive vaccine strains of the B/Victoria and B/Yamagata lineages are shown in red and blue, respectively. Genetic reassortants having B/Victoria lineage HA detected from 2002 to 2003 are indicated by red asterisks, and those having B/Yamagata lineage HA detected from 2014 to 2015 are indicated by blue asterisks. Black stars indicate the strains isolated in Japan in 2016 and after 2016. HA lineage is shown after each strain name.

We determined the sequences of the entire coding region of the B126-2 virus and nine reassortants, which were used to construct a phylogenetic tree. The sequences of each segment of the B126 viruses belonged to either the B/Phuket/3073/2013-like cluster or the B/Texas/02/2013-like cluster (Figure 2). The genomic compositions of the B126 viruses were consistent with those of the corresponding plaques (Figure 1B). When we analyzed some strains isolated in 2016 and after 2016 that had been registered in GISAID, no genetic reassortants were found in the Japanese B/Yamagata and B/Victoria strains, including those isolated in Yamagata Prefecture, and their genomic composition exactly matched the respective vaccine strains used since 2016, such as B/Phuket/3073/2013 (B/Yamagata), B/Texas/02/2013 (B/Victoria), B/Victoria/705/2018 (B/Victoria), and B/Austria/1359417/2021 (B/Victoria) (Figure 2).

### 3.4. Growth Kinetics of B126 Viruses in Cultured Cells

**Figure 3:**
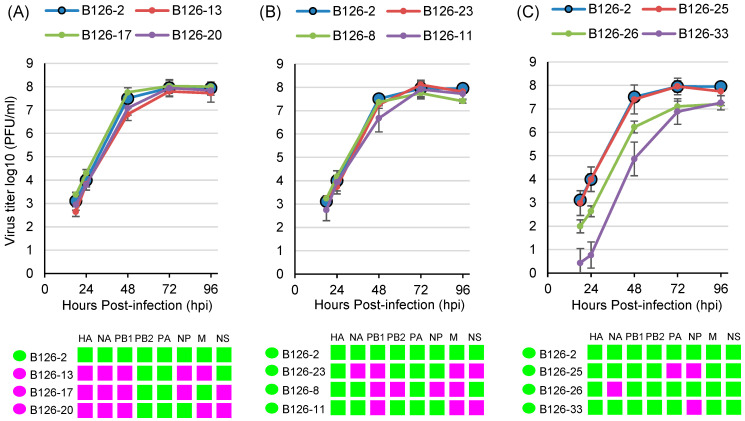
Replication kinetics of reassortants derived from B126 specimen on MDCK cells. The growth kinetics of B126-2 (all segments are B/Yamagata lineage) and the indicated reassortants, in which five segments had B/Victoria lineage genes (**A**), three or four segments had B/Victoria genes (**B**), and one or two segments had B/Victoria genes (**C**), were compared. The genomic composition of the reassortants at the lineage level is shown below the graph.

To examine the replicative fitness of the B126 viruses, we compared the growth kinetics of the B126-2 virus, possessing B/Yamagata lineage sequences in all segments, and the nine B126-derived reassortants grown in MDCK cells. Three viruses, in which five of eight segments, including the HA segment, had B/Victoria lineage sequences, showed a level of growth matching that of the B126-2 virus (Figure 3A). Meanwhile, three viruses with B/Victoria lineage sequences in three or four of their eight segments also showed a growth level matching that of the B126-2 virus (Figure 3B). The B126-25 virus with B/Victoria lineage sequences in PA and NP segments replicated as efficiently as the B126-2 virus; however, B126-26 and B126-33 viruses, which possessed B/Victoria lineage sequences only in the NA and NP segments, respectively, exhibited lower titers than the B126-2 virus (Figure 3C).

## 4. Discussion

In this study, we demonstrated coinfection with two influenza B viruses belonging to the B/Yamagata and B/Victorias lineages in a Japanese child. We also observed the occurrence of genetic reassortment in this patient. To the best of our knowledge, genetic reassortment between influenza B virus lineages due to their coinfection in humans has not been reported previously.

A previous study by Laurie et al. [16] using a ferret model of human influenza demonstrated the occurrence of viral interference between influenza B virus lineages when infections occurred 3 days apart. Primary infection with B/Phuket/3073/2013 (B/Yamagata) prevented infection 3 days later with B/Brisbane/60/2008 (B/Victoria) in three out of four ferrets, but coinfection and seroconversion occurred in only one ferret. It was reported that primary virus infections were cleared after 4–9 days, while challenge virus shedding was delayed and quickly cleared. This research using a ferret model suggests that in the case reported here involving a 9-year-old child, infection with B/Phuket/3073/2013-like virus (B/Yamagata) occurred first, followed by that with B/Texas/02/2013-like virus (B/Victoria).

In the abovementioned experiment using a ferret model [16], infectious virus shedding was evaluated using the copy numbers of HA genes; therefore, the frequency of gene reassortment was unclear. In our case, 21 of the 34 plaques (61.8%) were B/Yamagata lineage viruses lacking gene reassortment. The proportion of B/Victoria lineage genes differed among the segments, with PB1 segments having the highest (23.5%) and PB2 and PA having the lowest (2.9%). These differences may be associated with variations in the efficiency of genome packaging [19], although further research on this issue is needed.

Here, we used plaque purification to obtain cloned viruses. Reassortants with each genome composition were clearly present in the throat swab samples. However, growth in cultured cells could not be achieved for four of the thirty-four plaques. This may have been because the virion level in those plaques was very low or because the replication efficiency of viruses with such a genomic composition was poor. Four plaques that did not grow in cultured cells were reassortants with B/Victoria lineage genes only in the PB1, NP, or NS segments. Another plaque, B126-33, possessing B/Victoria lineage genes only in the NP segment, exhibited growth in cultured cells, albeit with a much lower growth efficiency. If only one of the eight segments contain B/Victoria lineage genes, the efficiency of growth may be reduced. In fact, B126-25′s growth efficiency recovered when genes of the B/Victoria lineage were used in NP and PA segments. Interestingly, we obtained five reassortants with B/Victoria lineage genes in the NA segment, but the growth of reassortant B126-26 with B/Victoria lineage genes only in NA was lower than that of the other reassortants. Although the reason for the decrease in growth efficiency of these reassortants was unclear, it seems that the genomic composition influences growth efficiency.

Based on a phylogenetic analysis of the eight genome segments of influenza B viruses isolated since 1980, Dudas et al. [10] reported that either completely B/Victoria or B/Yamagata lineage-derived PB1-PB2-HA complexes are continuously maintained, suggesting the importance of having a “pure” lineage of the PB1-PB2-HA complex for whole-genome fitness. Kim et al. [20] investigated reassortment compatibility between PB1 and PB2 genes using a plasmid-based replicon assay and reported that ribonucleoproteins (RNPs) with a heterologous lineage PB1-PB2 pair exhibited reduced polymerase activity compared with the wild-type RNPs harboring a homologous lineage pair. Among the thirteen reassortants that we obtained, eight have a “nonpure” lineage of the PB1-PB2-HA complex, while seven have a heterologous lineage PB1-PB2 pair (Figure 1B). All reassortant viruses with a heterologous lineage of the PB1-PB2-HA complex, except two with B/Victoria lineage genes only in PB1, exhibited the same growth level as the virus with B/Yamagata lineage genes in all segments. However, these reassortants have not spread since then. This suggests the existence of other factors allowing such gene reassortants to become epidemic strains in the human population, besides growth efficiency.

During 2002 and 2004, B/Victoria lineage strains, such as B/Brisbane/32/2002 and B/Yamagata/115/2003 (red asterisks in Figure 2), inherited the HA, PB1, PB2, and NP segments derived from B/Shandong/7/97-like viruses (B/Victoria) and the NA, PA, M, and NS segments derived from B/Johannesburg/05/99-like viruses (B/Yamagata), including B/Yamagata/K500/2001 and B/Houston/B69/2002 (Figure 2) [3,8,9]. Subsequently, in 2007 and 2008, B/Victoria lineage strains, such as B/Brisbane/60/2008, acquired the B/Yamagata lineage NP segment [11,15] and continued to be prevalent as B/Texas/02/2013-like viruses to this day. Meanwhile, during 2014 and 2015, B/Yamagata lineage strains such as B/Hawaii/04/2014 and B/Sapporo/5/2015 (blue asterisks in Figure 2) inherited the NA segment from the B/Victoria lineage strains, while B/Sapporo/5/2015 also inherited the NP segment. Although such gene reassortants have been detected in various regions, including Thailand [12], they have not been detected as subsequent epidemic strains. The gene reassortants analyzed here have various genome compositions derived from B/Phuket/3073/2013-like viruses and B/Texas/02/2013-like viruses, but none of them was confirmed as a strain that subsequently caused an epidemic. It appears that no reassortants in the patient’s throat swab samples exhibited better growth efficiency than B/Yamagata lineage viruses without genetic reassortment and could be transmitted from person to person.

We demonstrated that, against the background of the simultaneous spread of the B/Yamagata and B/Victoria lineage strains in a community, various gene reassortments occurred in a coinfected child. Reassortants with genomic compositions not associated with reduced growth efficiency may appear more than expected. These results suggest that simultaneous outbreaks of two influenza B virus lineages increase genetic diversity and could lead to the emergence of new epidemic strains.

## Figures and Tables

**Table 1:** Antigenicity of representative influenza B virus strains isolated in Yamagata, Japan, in 2016.

Virus Strain	Specimen Collection Date	HI Titers Using Antisera Against:			
		B/Phuket/3073/2013 (B/Yamagata)	B/Texas/02/2013 (B/Victoria)	A/California/7/2009 (A/H1pdm09)	A/Switzerland/9715293/2013 (A/H3)
B/Yamagata/9/2016	20 January	<10	1280	<10	<10
B/Yamagata/126/2016 (B126)	2 March	320	160	<10	<10
B/Yamagata/131/2016	17 March	320	20	<10	<10
B/Yamagata/191/2016	6 May	<10	2560	<10	<10
B/Yamagata/202/2016	3 June	320	20	<10	<10

**Table 2:** Number of lineages for each segment among 34 plaques derived from B126.

Lineage	HA	NA	PB1	PB2	PA	NP	M	NS
B/Phuket/3073/2013 (B/Yamagata)	31	29	26	33	33	28	29	29
B/Texas/02/2013 (B/Victoria)	3	5	8	1	1	6	5	5
% of B/Victoria	8.8	14.7	23.5	2.9	2.9	17.6	14.7	14.7

## Data Availability

Data are contained within the article.

## Supplementary Materials

The following supporting information can be downloaded at: https://www.mdpi.com/article/10.3390/v16060983/s1, Table S1: Accession numbers of influenza B virus sequences in GenBank or GISAID used for the phylogenetic tree analysis.
