# Supplementary material for: Genetic Reassortment in a Child Coinfected with Two Influenza B Viruses, B/Yamagata Lineage and B/Victoria-Lineage Strains
Source: Viruses. 2024 Jun 19;16(6):983. doi: 10.3390/v16060983 (PMC11209448; doi:10.3390/v16060983)
Supplement: Supplementary file 1 [file viruses-16-00983-s001.zip › viruses-3022881-supplementary.pdf]

Table S1. Accession numbers of influenza B virus sequences in GenBank or GISAID used for the phylogenetic tree analysis.

<table>
<tr><th rowspan="2">Strains | HA lineage |</th><th rowspan="2">collection date/year</th><th colspan="8">Accession numbers in GenBank or GISAID</th></tr>
<tr><th>HA</th><th>NA</th><th>PB1</th><th>PB2</th><th>PA</th><th>NP</th><th>M</th><th>NS</th></tr>
<tr><td>B/Victoria/02/1987 | Victoria |</td><td>1987</td><td>CY018757</td><td>CY018759</td><td>CY018763</td><td>CY018764</td><td>CY018762</td><td>CY018760</td><td>CY018758</td><td>CY018761</td></tr>
<tr><td>B/Yamagata/16/88 | Yamagata |</td><td>1988</td><td>CY018765</td><td>CY018767</td><td>CY018771</td><td>CY018772</td><td>CY018770</td><td>CY018768</td><td>CY018766</td><td>CY018769</td></tr>
<tr><td>B/Shangdong/7/97 | Victoria |</td><td>1997</td><td>AF486836</td><td>AY044172</td><td>AY044173</td><td>AF484967</td><td>AF486835</td><td>AY044169</td><td>AY044171</td><td>AY044170</td></tr>
<tr><td>B/Yamanashi/166/98 | Yamagata |</td><td>1998</td><td>CY019531</td><td>CY019533</td><td>CY019537</td><td>CY019538</td><td>CY019536</td><td>CY019534</td><td>CY019532</td><td>CY019535</td></tr>
<tr><td>B/Johannesburg/05/1999 | Yamagata |</td><td>1999</td><td>CY018613</td><td>CY018615</td><td>CY018619</td><td>CY018620</td><td>CY018618</td><td>CY018616</td><td>CY018614</td><td>CY018617</td></tr>
<tr><td>B/Akita/27/2001 | Victoria |</td><td>2001</td><td>CY018677</td><td>CY018679</td><td>CY018683</td><td>CY018684</td><td>CY018682</td><td>CY018680</td><td>CY018678</td><td>CY018681</td></tr>
<tr><td>B/Hong Kong/330/2001 | Victoria |</td><td>2001</td><td>CY018709</td><td>CY018711</td><td>CY018715</td><td>CY018716</td><td>CY018714</td><td>CY018712</td><td>CY018710</td><td>CY018713</td></tr>
<tr><td>B/Yamagata/K490/2001 | Yamagata |</td><td>2001-03-21</td><td>LC811901</td><td>AB120244</td><td>LC811908</td><td>LC811915</td><td>LC811922</td><td>LC811929</td><td>AB120262</td><td>AB120436</td></tr>
<tr><td>B/Yamagata/K500/2001 | Yamagata |</td><td>2001-03-24</td><td>LC811902</td><td>AB120245</td><td>LC811909</td><td>LC811916</td><td>LC811923</td><td>LC811930</td><td>AB120263</td><td>AB120437</td></tr>
<tr><td>B/Yamagata/K501/2001 | Yamagata |</td><td>2001-03-24</td><td>LC811903</td><td>AB120246</td><td>LC811910</td><td>LC811917</td><td>LC811924</td><td>LC811931</td><td>AB120264</td><td>AB120438</td></tr>
<tr><td>B/Johannesburg/69/2001 | Yamagata |</td><td>2001-06-12</td><td>CY019539</td><td>CY019541</td><td>CY019545</td><td>CY019546</td><td>CY019544</td><td>CY019542</td><td>CY019540</td><td>CY019543</td></tr>
<tr><td>B/Brisbane/32/2002 | Victoria |</td><td>2002</td><td>CY018701</td><td>CY018703</td><td>CY018707</td><td>CY018708</td><td>CY018706</td><td>CY018704</td><td>CY018702</td><td>CY018705</td></tr>
<tr><td>B/Houston/B69/2002 | Yamagata |</td><td>2002-03-21</td><td>CY018149</td><td>CY018151</td><td>CY018155</td><td>CY018156</td><td>CY018154</td><td>CY018152</td><td>CY018150</td><td>CY018153</td></tr>
<tr><td>B/Yamagata/222/2002 | Victoria |</td><td>2002</td><td>LC811898</td><td>AB120254</td><td>LC811905</td><td>LC811912</td><td>LC811919</td><td>LC811926</td><td>AB120272</td><td>AB120446</td></tr>
<tr><td>B/Yamagata/115/2003 | Victoria |</td><td>2003</td><td>LC811897</td><td>AB120255</td><td>LC811904</td><td>LC811911</td><td>LC811918</td><td>LC811925</td><td>AB120273</td><td>AB120447</td></tr>
<tr><td>B/Yamagata/1246/2003 | Yamagata |</td><td>2003</td><td>LC811899</td><td>AB120256</td><td>LC811906</td><td>LC811913</td><td>LC811920</td><td>LC811927</td><td>AB120274</td><td>AB120448</td></tr>
<tr><td>B/Yamagata/1311/2003 | Yamagata |</td><td>2003</td><td>LC811900</td><td>AB120257</td><td>LC811907</td><td>LC811914</td><td>LC811921</td><td>LC811928</td><td>AB120275</td><td>AB120449</td></tr>
<tr><td>B/Malaysia/2506/2004 | Victoria |</td><td>2004</td><td>CY040449</td><td>CY040451</td><td>CY040455</td><td>CY040456</td><td>CY040454</td><td>CY040452</td><td>CY040450</td><td>CY040453</td></tr>
<tr><td>B/Florida/4/2006 | Yamagata |</td><td>2006</td><td>CY033876</td><td>CY033878</td><td>CY033882</td><td>CY033883</td><td>CY033881</td><td>CY033879</td><td>CY033877</td><td>CY033880</td></tr>
<tr><td>B/Brisbane/60/2008 | Victoria |</td><td>2008-08-04</td><td>EPI902456</td><td>EPI902455</td><td>EPI903345</td><td>EPI903525</td><td>EPI902997</td><td>EPI902996</td><td>EPI902804</td><td>EPI903705</td></tr>
<tr><td>B/Massachusetts/02/2012 | Yamagata |</td><td>2012-03-13</td><td>EPI904336</td><td>EPI904569</td><td>EPI905734</td><td>EPI905987</td><td>EPI905501</td><td>EPI905268</td><td>EPI904802</td><td>EPI905035</td></tr>
<tr><td>B/Texas/02/2013 | Victoria |</td><td>2013-01-09</td><td>EPI443690</td><td>EPI443689</td><td>EPI443688</td><td>EPI443687</td><td>EPI443686</td><td>EPI447191</td><td>EPI447193</td><td>EPI447192</td></tr>
<tr><td>B/Phuket/3073/2013 | Yamagata |</td><td>2013-11-21</td><td>EPI544264</td><td>EPI544263</td><td>EPI544262</td><td>EPI544261</td><td>EPI547694</td><td>EPI544260</td><td>EPI592901</td><td>EPI547693</td></tr>
<tr><td>B/Hawaii/04/2014 | Yamagata |</td><td>2014-03-19</td><td>EPI531138</td><td>EPI531137</td><td>EPI541663</td><td>EPI541664</td><td>EPI541662</td><td>EPI541661</td><td>EPI553977</td><td>EPI531136</td></tr>
<tr><td>B/Hawaii/38/2014 | Yamagata |</td><td>2014-12-31</td><td>EPI568903</td><td>EPI568902</td><td>EPI568901</td><td>EPI568900</td><td>EPI568899</td><td>EPI568897</td><td>EPI575930</td><td>EPI568898</td></tr>
<tr><td>B/Sapporo/5/2015 | Yamagata |</td><td>2015-02-23</td><td>EPI585068</td><td>EPI585067</td><td>EPI687924</td><td>EPI687923</td><td>EPI687922</td><td>EPI687919</td><td>EPI687921</td><td>EPI687920</td></tr>
<tr><td>B/Yamagata/77/2015 | Yamagata |</td><td>2015-04-23</td><td>EPI628882</td><td>EPI628881</td><td>EPI688014</td><td>EPI688013</td><td>EPI688012</td><td>EPI688009</td><td>EPI688011</td><td>EPI688010</td></tr>
<tr><td>B/Yamagata/9/2016 | Victoria |</td><td>2016-01-20</td><td>LC811811</td><td>LC811871</td><td>LC811823</td><td>LC811835</td><td>LC811847</td><td>LC811859</td><td>LC811883</td><td>LC811895</td></tr>
<tr><td>B/Yamagata/131/2016 | Yamagata |</td><td>2016-03-17</td><td>LC811812</td><td>LC811872</td><td>LC811824</td><td>LC811836</td><td>LC811848</td><td>LC811860</td><td>LC811884</td><td>LC811896</td></tr>
<tr><td>B/Yamagata/191/2016 | Victoria |</td><td>2016-05-06</td><td>EPI824372</td><td>EPI824371</td><td>EPI840178</td><td>EPI840177</td><td>EPI840176</td><td>EPI840173</td><td>EPI840175</td><td>EPI840174</td></tr>
<tr><td>B/Yamagata/202/2016 | Yamagata |</td><td>2016-06-03</td><td>EPI824582</td><td>EPI824581</td><td>EPI824580</td><td>EPI824579</td><td>EPI824578</td><td>EPI824575</td><td>EPI824577</td><td>EPI824576</td></tr>
<tr><td>B/Maryland/15/2016 | Victoria |</td><td>2016-12-27</td><td>EPI1255272</td><td>EPI1255271</td><td>EPI1255270</td><td>EPI1255269</td><td>EPI1255268</td><td>EPI1255265</td><td>EPI1255267</td><td>EPI1255266</td></tr>
<tr><td>B/Kanagawa/AC1623/2017 | Victoria |</td><td>2017-01-17</td><td>EPI909630</td><td>EPI909629</td><td>EPI909628</td><td>EPI909627</td><td>EPI909626</td><td>EPI909623</td><td>EPI909625</td><td>EPI909624</td></tr>
<tr><td>B/Colorado/06/2017 | Victoria |</td><td>2017-02-25</td><td>EPI1056637</td><td>EPI1056636</td><td>EPI1269609</td><td>EPI1269610</td><td>EPI1269608</td><td>EPI1056633</td><td>EPI1056635</td><td>EPI1056634</td></tr>
<tr><td>B/Kanagawa/IC1649/2017 | Yamagata |</td><td>2017-03-22</td><td>EPI975702</td><td>EPI975701</td><td>EPI1054234</td><td>EPI1054233</td><td>EPI1054232</td><td>EPI1054229</td><td>EPI1054231</td><td>EPI1054230</td></tr>
<tr><td>B/Yamagata/103/2017 | Yamagata |</td><td>2017-04-14</td><td>EPI1027334</td><td>EPI1027333</td><td>EPI1027332</td><td>EPI1027331</td><td>EPI1027330</td><td>EPI1027327</td><td>EPI1027329</td><td>EPI1027328</td></tr>
<tr><td>B/Hong Kong/269/2017 | Victoria |</td><td>2017-05-24</td><td>EPI1052656</td><td>EPI1052655</td><td>EPI1052654</td><td>EPI1052653</td><td>EPI1052652</td><td>EPI1052649</td><td>EPI1052651</td><td>EPI1052650</td></tr>
<tr><td>B/Yamagata/19/2018 | Yamagata |</td><td>2018-01-15</td><td>EPI1223615</td><td>EPI1223614</td><td>EPI1223613</td><td>EPI1223612</td><td>EPI1223611</td><td>EPI1223608</td><td>EPI1223610</td><td>EPI1223609</td></tr>
<tr><td>B/Yokohama/86/2018 | Victoria |</td><td>2018-04-08</td><td>EPI1244713</td><td>EPI1244712</td><td>EPI1244711</td><td>EPI1244710</td><td>EPI1244709</td><td>EPI1244706</td><td>EPI1244708</td><td>EPI1244707</td></tr>
<tr><td>B/Victoria/705/2018 | Victoria |</td><td>2018-10-30</td><td>EPI1629575</td><td>EPI1629574</td><td>EPI1629573</td><td>EPI1629572</td><td>EPI1629571</td><td>EPI1629568</td><td>EPI1629570</td><td>EPI1629569</td></tr>
<tr><td>B/Washington/2/2019 | Victoria |</td><td>2019-01-19</td><td>EPI2413715</td><td>EPI2413713</td><td>EPI2413717</td><td>EPI2413719</td><td>EPI2413714</td><td>EPI2413716</td><td>EPI2413720</td><td>EPI2413718</td></tr>
<tr><td>B/Kanagawa/IC18169/2019 | Victoria |</td><td>2019-04-27</td><td>EPI1592960</td><td>EPI1592959</td><td>EPI1592958</td><td>EPI1592957</td><td>EPI1592956</td><td>EPI1592953</td><td>EPI1592955</td><td>EPI1592954</td></tr>
<tr><td>B/Yokohama/9/2019 | Yamagata |</td><td>2019-05-02</td><td>EPI1592992</td><td>EPI1592991</td><td>EPI1592990</td><td>EPI1592989</td><td>EPI1592988</td><td>EPI1592985</td><td>EPI1592987</td><td>EPI1592986</td></tr>
<tr><td>B/Yamagata/162/2019 | Victoria |</td><td>2019-06-12</td><td>EPI1589322</td><td>EPI1589321</td><td>EPI1589320</td><td>EPI1589319</td><td>EPI1589318</td><td>EPI1589315</td><td>EPI1589317</td><td>EPI1589316</td></tr>
<tr><td>B/Yokohama/1/2020 | Victoria |</td><td>2020-01-08</td><td>EPI1799722</td><td>EPI1799721</td><td>EPI1799720</td><td>EPI1799719</td><td>EPI1799718</td><td>EPI1799715</td><td>EPI1799717</td><td>EPI1799716</td></tr>
<tr><td>B/Yamagata/29/2020 | Victoria |</td><td>2020-01-21</td><td>EPI1766744</td><td>EPI1766743</td><td>EPI1766742</td><td>EPI1766741</td><td>EPI1766740</td><td>EPI1766737</td><td>EPI1766739</td><td>EPI1766738</td></tr>
<tr><td>B/Austria/1359417/2021 | Victoria |</td><td>2021-01-09</td><td>EPI2413502</td><td>EPI2413500</td><td>EPI2413504</td><td>EPI2413506</td><td>EPI2413501</td><td>EPI2413503</td><td>EPI2413507</td><td>EPI2413505</td></tr>
<tr><td>B/Yokohama/1/2023 | Victoria |</td><td>2023-02-24</td><td>EPI2562967</td><td>EPI2562966</td><td>EPI2562965</td><td>EPI2562964</td><td>EPI2562963</td><td>EPI2562960</td><td>EPI2562962</td><td>EPI2562961</td></tr>
<tr><td>B126-2 (B/Yamagata/126C2/2016) | Yamagata |</td><td>2016-03-02</td><td>LC811801</td><td>LC811861</td><td>LC811813</td><td>LC811825</td><td>LC811837</td><td>LC811849</td><td>LC811873</td><td>LC811885</td></tr>
<tr><td>B126-8 (B/Yamagata/126C8/2016) | Yamagata |</td><td>2016-03-02</td><td>LC811802</td><td>LC811862</td><td>LC811814</td><td>LC811826</td><td>LC811838</td><td>LC811850</td><td>LC811874</td><td>LC811886</td></tr>
<tr><td>B126-11 (B/Yamagata/126C11/2016) | Yamagata |</td><td>2016-03-02</td><td>LC811803</td><td>LC811863</td><td>LC811815</td><td>LC811827</td><td>LC811839</td><td>LC811851</td><td>LC811875</td><td>LC811887</td></tr>
<tr><td>B126-13 (B/Yamagata/126C13/2016) | Victoria |</td><td>2016-03-02</td><td>LC811804</td><td>LC811864</td><td>LC811816</td><td>LC811828</td><td>LC811840</td><td>LC811852</td><td>LC811876</td><td>LC811888</td></tr>
<tr><td>B126-17 (B/Yamagata/126C17/2016) | Victoria |</td><td>2016-03-02</td><td>LC811805</td><td>LC811865</td><td>LC811817</td><td>LC811829</td><td>LC811841</td><td>LC811853</td><td>LC811877</td><td>LC811889</td></tr>
<tr><td>B126-20 (B/Yamagata/126C20/2016) | Victoria |</td><td>2016-03-02</td><td>LC811806</td><td>LC811866</td><td>LC811818</td><td>LC811830</td><td>LC811842</td><td>LC811854</td><td>LC811878</td><td>LC811890</td></tr>
<tr><td>B126-23 (B/Yamagata/126C23/2016) | Yamagata |</td><td>2016-03-02</td><td>LC811807</td><td>LC811867</td><td>LC811819</td><td>LC811831</td><td>LC811843</td><td>LC811855</td><td>LC811879</td><td>LC811891</td></tr>
<tr><td>B126-25 (B/Yamagata/126C25/2016) | Yamagata |</td><td>2016-03-02</td><td>LC811808</td><td>LC811868</td><td>LC811820</td><td>LC811832</td><td>LC811844</td><td>LC811856</td><td>LC811880</td><td>LC811892</td></tr>
<tr><td>B126-26 (B/Yamagata/126C26/2016) | Yamagata |</td><td>2016-03-02</td><td>LC811809</td><td>LC811869</td><td>LC811821</td><td>LC811833</td><td>LC811845</td><td>LC811857</td><td>LC811881</td><td>LC811893</td></tr>
<tr><td>B126-33 (B/Yamagata/126C33/2016) | Yamagata |</td><td>2016-03-02</td><td>LC811810</td><td>LC811870</td><td>LC811822</td><td>LC811834</td><td>LC811846</td><td>LC811858</td><td>LC811882</td><td>LC811894</td></tr>
</table>
